# Supplementary material for: microRNAs Control Antiviral Immune Response, Cell Death and Chemotaxis Pathways in Human Neuronal Precursor Cells (NPCs) during Zika Virus Infection
Source: Int J Mol Sci. 2022 Sep 7;23(18):10282. doi: 10.3390/ijms231810282 (PMC9499039; doi:10.3390/ijms231810282)
Supplement: Supplementary file 1 [file ijms-23-10282-s001.zip › ijms-1831073-supplementary.pdf]

Supplementary Materials:

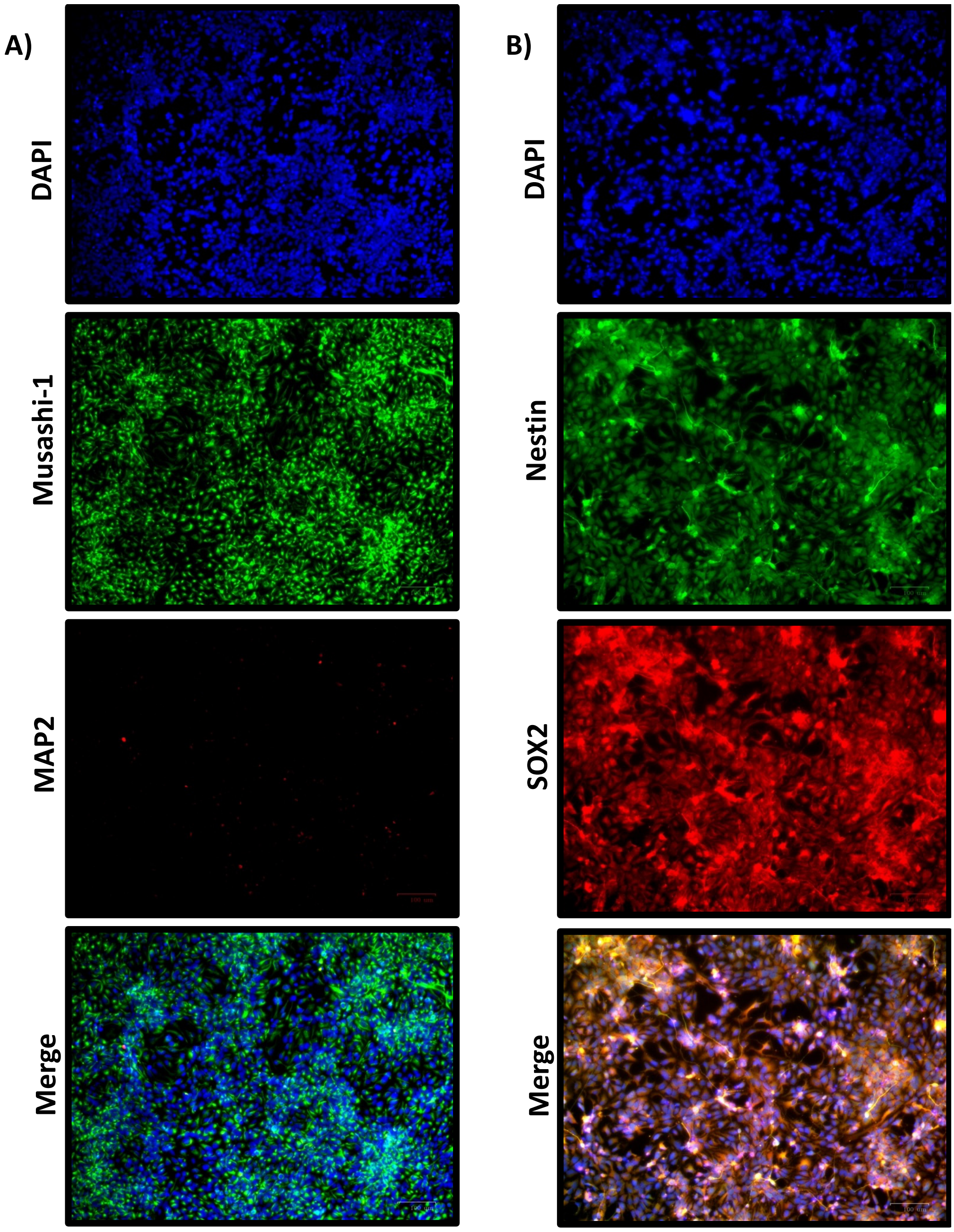

Figure S1. Characterization of human NPCs. Immunofluorescence analysis of NPCs-specific markers. **A)** DAPI (blue), Musashi-1 (green), MAP2 (red) and merge images. **B)** DAPI (blue), Nestin (green), SOX2 (red) and merge images. 20X magnification.
